# Discovery of *bis*-thiourea derivatives as potent tyrosinase inhibitors: combined experimental and computational study

**DOI:** 10.1080/14756366.2025.2518195

**Published:** 2025-06-24

**Authors:** Sahachai Sabuakham, Sutita Nasoontorn, Napat Nuramrum, Atit Silsirivanit, Thanyada Rungrotmongkol, Ratchanok Pingaew, Panupong Mahalapbutr

**Affiliations:** a Department of Biochemistry, Center for Translational Medicine, Faculty of Medicine, Khon Kaen University, Khon Kaen, Thailand; b Program in Bioinformatics and Computational Biology, Graduate School, Chulalongkorn University, Bangkok, Thailand; c Center of Excellence in Structural and Computational Biology, Department of Biochemistry, Faculty of Science, Chulalongkorn University, Bangkok, Thailand; d Department of Chemistry, Faculty of Science, Srinakharinwirot University, Bangkok, Thailand

**Keywords:** *Bis*-thioureas, computational biology, molecular docking, molecular dynamics simulations, tyrosinase

## Abstract

Tyrosinase, a key enzyme in melanin synthesis, serves as a primary target for developing depigmenting agents. The search for novel tyrosinase inhibitors is needed due to the adverse effects of current inhibitors. This study evaluated 16 *bis*-thiourea derivatives using *in vitro* and *in silico* methods, identifying compound **4**, with chlorine substituents, as the most potent inhibitor. Compound **4** outperformed kojic acid in inhibiting mushroom tyrosinase activity and interacted with catalytic copper ions and active site residues, as revealed by molecular docking and copper-chelating assay. Molecular dynamics simulation and MM/PBSA-based free energy calculations confirmed the greater stability and binding affinity of the compound **4**-tyrosinase complex in an aqueous environment compared to kojic acid-tyrosinase complex. Melanin assay revealed that compound **4** significantly suppressed melanin production in B16F10 melanoma cells, showing stronger anti-melanogenic activity than kojic acid. Drug-likeness predictions confirmed its compliance with Lipinski’s rule of five, supporting *bis*-thiourea derivatives as promising tyrosinase inhibitors.

## Graphical Abstract

**Figure:**
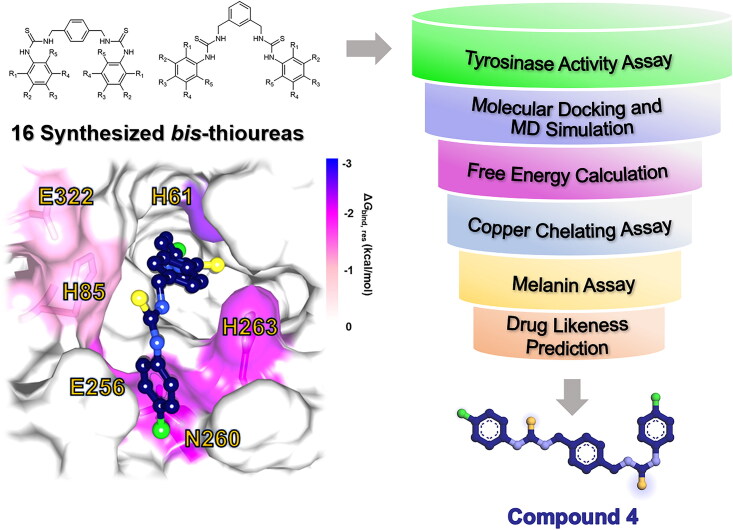

## 1. Introduction

**Figure 1.**
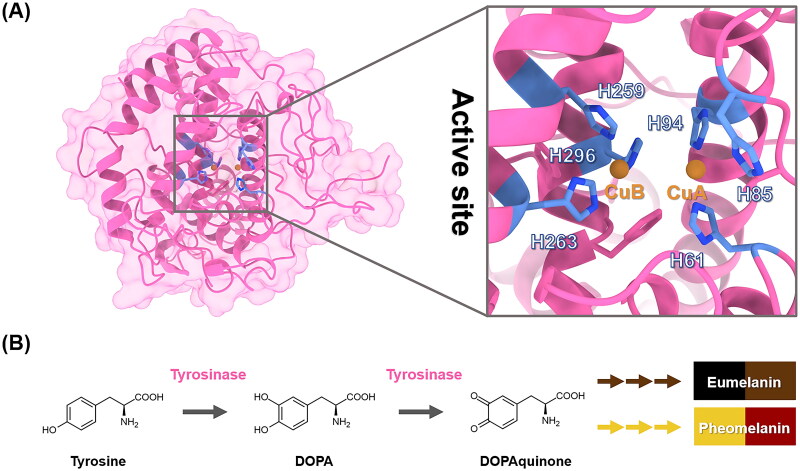
(**A**) Crystal structure of tyrosinase from *Agaricus bisporus* (PDB ID: 2Y9X)^17^. The catalytic histidine residues and copper ions (CuA and CuB) were labelled in cornflower blue and orange, respectively. (**B**) Melanogenesis pathway (production of eumelanin and pheomelanin).

Melanogenesis is a multi-step process that occurs in the melanosome of melanocytes, leading to the formation of the biopolymer known as melanin^1^. Tyrosinase, a membrane-bound enzyme located on the melanosome of melanocytes^2^, catalyses the rate-limiting step of melanogenesis^2–5^ (Figure 1A) by converting l-tyrosine to l-DOPA, leading to melanin production^6^ (Figure 1B). Eumelanin contributes to black and brown pigmentation, whereas pheomelanin is responsible for red and yellow tones^7^^,^^8^. Nevertheless, excessive melanin production can result in hyperpigmentation disorders such as melasma, lentigines, and freckles^9^. Although there are clinically available tyrosinase inhibitors, most of them exhibit adverse side effects^8^. For instance, hydroquinone and arbutin have been reported to cause skin irritation and contact dermatitis^10^^,^^11^. Therefore, there is a need to discover novel tyrosinase inhibitors with improved potency and safety profiles.

Previous studies have shown that several thiourea derivatives could act as potent tyrosinase inhibitors. The *N*-aryl-*N′*-substituted phenylthiourea derivatives, especially compound bearing trifluoromethyl moiety exhibited tyrosinase inhibitory activity with an IC_50_ value of 6.13 μM^12^. The thiourea-containing drugs, thioacetazone and ambazone, can suppress tyrosinase activity with IC_50_ values of 14 and 15 µM, respectively^13^. Phenylthiourea (PTU) has been reported as a potent tyrosinase inhibitor^14^^,^^15^, and could induce tyrosinase degradation^14^. Furthermore, the combination of PTU with linderanolide B showed a synergistic effect on tyrosinase inhibition^16^.

Although the tyrosinase inhibitory activity of several thiourea derivatives has been investigated, there are no reports on the anti-tyrosinase potential of *bis*-thiourea derivatives. Based on the previous reports mentioned above, we hypothesise that the *bis*-thioureas could act as tyrosinase inhibitors.

In this study, we investigated the inhibitory potential of 16 *bis*-thiourea derivatives (Table 1) against tyrosinase and used kojic acid (KA) as a positive control through a combination of *in vitro* and *in silico* approaches. Initial identification of potent compounds was achieved using a mushroom tyrosinase activity assay. Molecular docking, molecular dynamics (MD) simulation, and free energy calculation based on the molecular mechanics/Poisson–Boltzmann surface area (MM/PBSA) method were subsequently employed to elucidate the binding mechanism of the screened *bis*-thiourea against tyrosinase at the atomic level. Furthermore, copper chelation and melanin assays were utilised to evaluate the copper-chelating potential and the anti-melanogenic activity of the screened compound.

**Table 1.** Chemical structures of 16 synthesised *bis*-thiourea derivatives^18–20^.

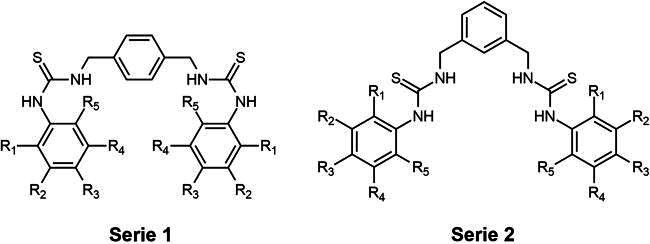					
Compound	R_1_	R_2_	R_3_	R_4_	R_5_
**Serie 1**					
**1**	H	H	CH_3_	H	H
**2**	H	H	OCH_3_	H	H
**3**	H	H	F	H	H
**4**	H	H	Cl	H	H
**5**	H	H	Br	H	H
**6**	H	H	NO_2_	H	H
**7**	H	H	CF_3_	H	H
**8**	H	CF_3_	H	CF_3_	H
**Serie 2**					
**9**	H	H	CH_3_	H	H
**10**	H	H	OCH_3_	H	H
**11**	H	H	F	H	H
**12**	H	H	Cl	H	H
**13**	H	H	Br	H	H
**14**	H	H	NO_2_	H	H
**15**	H	H	CF_3_	H	H
**16**	H	CF_3_	H	CF_3_	H

## 2. Methods and materials

### 2.1. Purity of bis-thioureas (1–16)

The 16 *bis*-thiourea derivatives (Table 1) were synthesised following the procedure. A mixture of xylylenediamine (2 mmol) and appropriate phenylisothiocyanate (4 mmol) in dichloromethane (20 ml) was stirred at room temperature for 3–16 h. The reaction was monitored by TLC, and then the solid product formed was filtered. The crude product was recrystallized to obtain the *bis*-thiourea derivatives **1**–**16**. Their NMR data have been reported in our previous work^18–20^. In this study, compound purities were determined by High Performance Liquid Chromatography (HPLC). HPLC was performed by Waters, 600 pump and controller, 717 autosampler, equipped with 996 PDA detector at 254 nm, column (Nova-Pak, C18, 4 μm, 60 Å, 150 mm × 3.9 mm), using isocratic 60:40 (acetonitrile:H_2_O), flow rate 1 ml/min, injection volume 5.0 μL. Each sample was prepared in acetonitrile/H_2_O (9/1).

### 2.2. Chemical reagent and mushroom tyrosinase activity assay

The procedure for the mushroom tyrosinase activity assay was conducted in accordance with the methodology described in a previous report^21^. KA was purchased from Sigma Aldrich (St. Louis, MO, USA). In a 96-well plate, 40 μL of mushroom tyrosinase (5 U/μL, Sigma Aldrich, St. Louis, MO, USA) was added to each well, followed by 50 μL of the test compound at various concentrations. After that, 10 μL of 50 mM l-DOPA (purity ≥98%, Sigma-Aldrich, St. Louis, MO, USA) were added and incubated in darkness at 37 °C for 15 min. Finally, the absorbance was measured at 450 nm. The experiment was conducted in triplicate. IC_50_ values were determined using GraphPad Prism 10.2.2 software.

### 2.3. System preparation and molecular docking

The 3D crystal structure of tyrosinase from *Agaricus bisporus* was retrieved from the Protein Data Bank with PDB ID: 2Y9X^17^. The protonation state of tyrosinase was assessed at a pH of 7.4 using the PDB2PQR web server^22^. The covalent thioether bond between the carbon atom of H85 and the sulphur atom of C83 was constructed as previously described^17^. The 3D structures of ligands were constructed using the GaussView 6.1 program^23^. The protonation state of ligands was assessed at a pH of 7.4 using the Marvinsketch program software^24^. According to standard protocols^25–29^, the electrostatic potential (ESP) charges of each ligand was calculated using the HF/6-31G(d) level of theory through the Gaussian 09^30^. The resonance structure of *bis*-thiourea moiety after optimisation agreed well with previous reports^31^^,^^32^. Molecular docking was conducted using CB-DOCK2 software^33^. The interaction profile of the protein–ligand complex was analysed using the BIOVIA Discovery Studio Visualiser. The docked complexes with the lowest docking energy were chosen for MD simulation.

### 2.4. MD simulation

The all-atom MD simulation of each complex was performed using the AMBER16 software package^34^. The parameters for both bonded and non-bonded interactions of all ligands were handled using the General Amber Force Field (GAFF)^35^. The protein parameters were defined using the AMBER ff14SB force field^36^. The missing hydrogen atoms were added using the LEaP module. Each system was solvated using TIP3P water molecules^37^. To maintain neutrality, sodium ions were included. In the isobaric–isothermal (*NPT*) ensemble, a boundary condition was set with a constant pressure of 1 atm and a temperature of 310 K. The SHAKE algorithm^38^ was employed to constrain hydrogen bonds, and a time step of 2 fs was used. Non-bonded interactions were computed with a residue-based cut-off of 12 Å, and the particle mesh Ewald method^39^ was utilised for handling long-range electrostatic interactions. To eliminate unfavourable contacts, the added hydrogen atoms were subsequently minimised using 1000 iterations of the steepest descent (SD) method, followed by 2000 iterations of conjugated gradient (CG) energy minimisation. Subsequently, the entire system was gradually heated to 310 K over 100 ps. The systems underwent restrained MD simulations for a total of 5.0 ns, with progressively decreasing restraints of 50, 30, 20, 10, 5, and 1 kcal/mol·Å^2^. Afterward, the systems were equilibrated for 500 ps. Finally, the MD simulation was performed without any restraints in the *NPT* ensemble (1 atm and 310 K) until reaching 40 ns. The MD simulation snapshots taken between 30 and 40 ns were selected for further analysis.

### 2.5. Structural analysis and binding free energy calculation

The CPPTRAJ module^40^ of AMBER16 was employed to calculate structural and dynamic properties, including root-mean-square deviation (RMSD), the number of atom contacts (#Contacts), radius of gyration (Rg), hydrogen bond (H-bond) occupation was analysed for the last 10 ns of simulation. Additionally, the stabilising amino acid residues in the binding process and the binding free energy (Δ*G*_bind_) of the ligand–protein complexes were determined using the MM/PBSA method^41^^,^^42^, with an interior dielectric constant of 5.0 and without normal mode analysis using 100 snapshots extracted from the final 10 ns of the MD simulation.

### 2.6. Drug-likeness prediction

Drug-likeness properties of all studied ligands were analysed using the SwissADME web tool (www.swissadme.ch)^43^.

### 2.7. Copper-chelating activity assay

The copper-chelating activity of compound **4** was assessed using previously reported protocol^44^ with minor modifications. Briefly, 40 μL of CuSO_4_ (1.25 mM) was added to each well of a 96-well plate containing 10 μL of pyrocatechol violet (4 mM), 40 μL of tested compound (**4**, KA, or EDTA; final concentration: 5 mM), and 10 μL of sodium acetate buffer (50 mM, pH 6.0). After that, the absorbance of each well was measured at 620 nm. The copper-chelating activity was calculated using the formula: Copper-chelating activity (%) = [(*A*_con_ – *A*_sam_)/*A*_con_] × 100, where *A*_con_ and *A*_sam_ are the absorbance of the control and test sample, respectively.

### 2.8. Cytotoxicity assay

Cell viability was determined using the MTT assay (3-(4,5-dimethylthiazol-2-yl)-2,5-diphenyltetrazolium bromide). B16F10 (murine melanoma cell line) was purchased from American Type Culture Collection (ATCC, Manassas, VA, USA). B16F10 cells were seeded into 96-well plates at a density of 1500 cells per well. After overnight incubation, the cells were treated with different concentrations of the test compound for 48 h. Following this, MTT reagent was added to each well and incubated for 3 h. The culture medium was then removed, and 100 μL of DMSO was used to dissolve the formazan crystals. Absorbance was measured at 540 nm to evaluate cell viability.

### 2.9. Melanin assay

Melanin production was assessed according to the methodology outlined in a previous study^45^. B16F10 cells were cultured in 6-well plates at a density of 100,000 cells per well. After overnight incubation, the cells were treated with different concentrations of the test compound for 48 h. Note that 1 μM of α-melanocyte-stimulating hormone (α-MSH) (Sigma Aldrich, St. Louis, MO, USA) was used to induce melanin synthesis. Following treatment, the cells were washed twice with PBS, detached using trypsin/EDTA, and centrifuged at 3,000×*g* for 5 min. The resulting cell pellets, containing approximately 700,000 cells, were solubilised in 2 M NaOH and heated at 100 °C for 30 min. Melanin content was quantified by measuring absorbance at 405 nm.

### 2.10. Statistical analysis

Data are presented as the mean ± standard error of the mean (SEM) from three independent experiments (*n* = 3). Statistical comparisons between two groups were conducted using the *t* test. A *p* values of <0.05 was considered statistically significant.

## 3. Results and discussion

### 3.1. Synthesis of bis-thiourea derivatives

The 16 in-house *bis*-thiourea derivatives were prepared by reaction of *m*- or *p*-xylylenediamine with the corresponding phenylisothiocyanate. The detailed characterisation of these compounds was provided in our previous work^18–20^. All biological evaluated compounds **1**–**16** were >95% purity by HPLC analysis (Figures S1–S^16^).

### 3.2. Effect of bis-thioureas on tyrosinase activity

**Figure 2.**
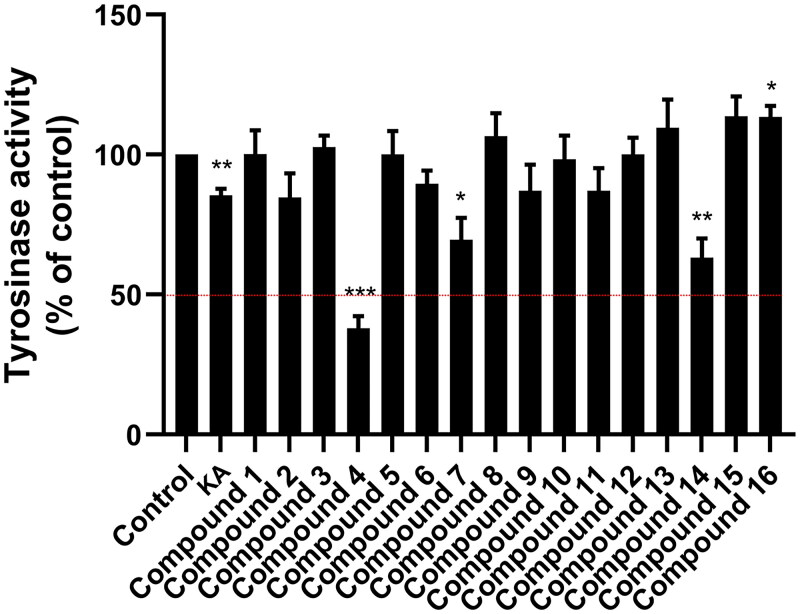
Tyrosinase inhibitory activity of 16 *bis*-thiourea derivatives and KA at a concentration of 50 μM. l-DOPA was used as a substrate. Data are shown as the mean ± standard error of the mean (SEM) (*n* = 3). *p < 0.05, **p < 0.01, ***p < 0.001 vs. control.

Initially, we screened for the most potent tyrosinase inhibitors from 16 synthesised *bis*-thiourea derivatives at a concentration of 50 μM. Figure 2 illustrated that compounds **4** (37.99 ± 4.29%), **14** (63.24 ± 6.85%), and **7** (69.60 ± 7.89%) exhibited higher tyrosinase inhibitory activity than the other derivatives and KA. Based on this finding, the most potent compound **4** together with KA were selected to determine their half-maximal inhibitory concentration (IC_50_).

**Figure 3.**
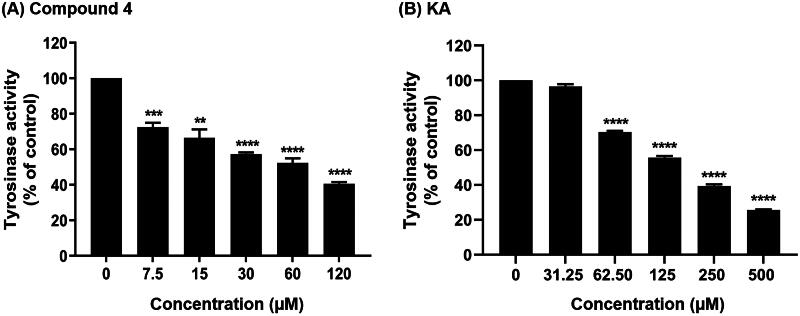
Tyrosinase inhibitory activity of (**A**) compound **4** and (**B**) KA at various concentrations. l-DOPA was used as a substrate. Data are shown as the mean ± SEM (*n* = 3). **p < 0.01, ***p < 0.001, ****p < 0.0001. vs. control.

As shown in Figure 3 and Table 2, compound **4**, containing chlorine substituents on the aromatic moieties, exhibited a higher tyrosinase inhibitory potential (IC_50_ = 61.63 ± 7.82 μM) than KA (IC_50_ = 168.73 ± 3.24 μM). Note that our IC_50_ value for KA aligns closely with previously reported IC_50_ values, which range from 235 to 250 μM^46^^,^^47^. Interestingly, compound **4** exhibited better inhibitory potency against tyrosinase compared to anisaldehyde (IC_50_ = 160 μM)^48^, amphotericin B (IC_50_ = 263.36 ± 11.76 μM)^45^, methimazole derivative (IC_50_ = 4100 μM)^49^, and glabrene (IC_50_ = 7600 μM)^50^, highlighting its potential for further development as a novel tyrosinase inhibitor. This finding was strongly supported by the structural stability, the compactness of the ligand–protein complex, the number of atom contacts (Figure 5), as well as binding affinity calculation (Table 3), which was discussed later.

**Figure 4.**
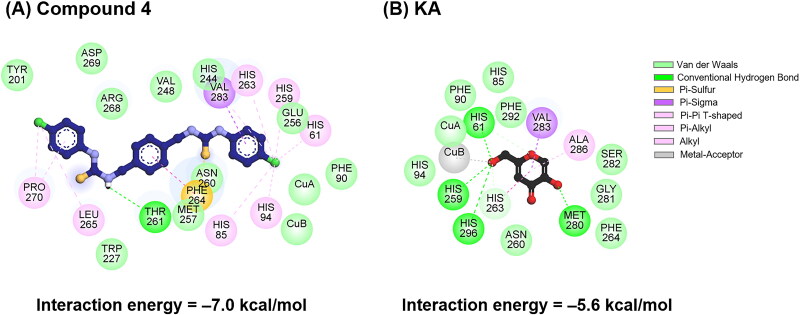
2D interaction profiles of (**A**) compound **4** and (**B**) KA in complexes with tyrosinase, obtained from molecular docking.

**Table 2.** IC_50_ of compound **4** and KA against mushroom tyrosinase.

Compound	IC_50_ (μM)
**4**	61.63 ± 7.82
KA	168.73 ± 3.24

**Table 3.** Δ*G*_bind_ and its energy components (kcal/mol) of compound **4**-tyrosinase and KA–tyrosinase complexes calculated with the MM/PBSA method.

Energy component	Compound 4-tyrosinase	KA-tyrosinase
**Δ*E*_vdW_**	–36.73 ± 0.44	–16.47 ± 0.32
**Δ*E*_ele_**	0.19 ± 0.17	–0.82 ± 0.24
**Δ*E*_MM_**	–36.54 ± 0.52	–17.29 ± 0.33
**Δ*G*_solv, nonpolar_**	–6.37 ± 0.05	–2.84 ± 0.03
**Δ*G*_solv, polar_**	8.19 ± 0.20	4.69 ± 0.16
**Δ*G*_solv_**	1.82 ± 0.17	1.84 ± 0.14
**Δ*G*_bind_**	–34.72 ± 0.45	–15.44 ± 0.38

Data are shown as the mean ± SEM from 100 snapshots extracted from the last 10 ns simulations.

Taken together, the most potent compound **4** was selected for further analyses, including (i) molecular docking, (ii) MD simulation, (iii) Δ*G*_bind_ calculation, (iv) H-bond occupation, (v) key binding residues, (vi) copper chelation activity assay, (vii) melanin assay, and (viii) drug-likeness prediction.

### 3.3. Molecular docking

The interaction energy and binding mode between tyrosinase and compound **4** was predicted using the CB-DOCK2 server^33^ as described in a previous study^51^. To validate the accuracy of this server, redocking was performed, and we found that the RMSD between the native ligand and the docked structure was 2.52 Å (Figure S17), which lies within the acceptable range of 2–3 Å^52^.

The molecular docking results showed that the interaction energy of compound **4** (–7.0 kcal/mol) was higher than that of KA (–5.6 kcal/mol), and both compounds could bind to the catalytic centre of tyrosinase. For the compound **4**-tyrosinase complex (Figure 4A), the hydrogen atom of its thiourea group could form H-bond with the T261 residue. The aromatic rings and chlorine atoms of compound **4** engaged in several Pi interactions with H61, H85, H94, H259, H263, F264, L265, P270, and V283 residues. Notably, van der Waals (vdW) interactions were found to mainly stabilise the complex formation between ligands/coppers and tyrosinase at the catalytic site, similar to the binding of luteolin, luteolin 5-*O*-*β*-d-glucopyranoside^53^, kuwanon G, and mulberrofuran G^54^ to tyrosinase.

**Figure 5.**
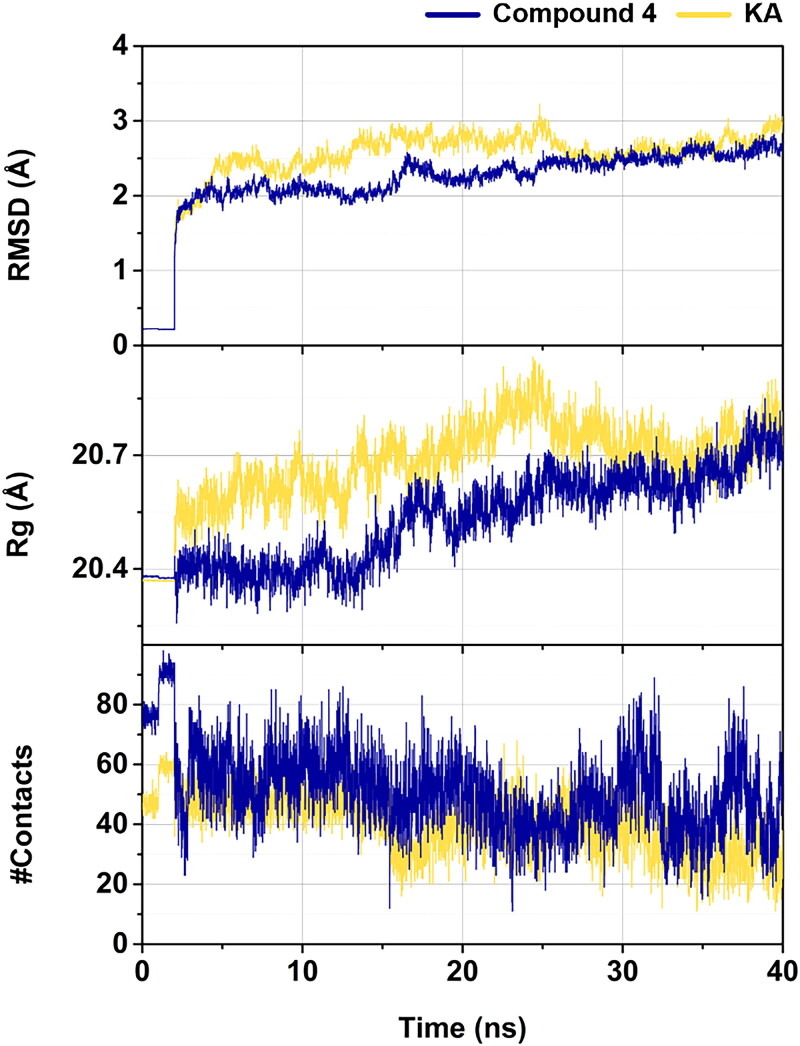
Time evolution of RMSD, radius of gyration (Rg), and number of contacts (#Contacts) for compound **4**–tyrosinase and KA–tyrosinase complexes.

In the case of the KA-tyrosinase system (Figure 4B), H-bond formations were detected between the hydroxyl (–OH) group of KA and H61, H259, M280, and H296 residues. In addition, Pi interactions and metal acceptor were detected at (i) V283 and A286 residues and (ii) CuB, respectively. It should be noted that the –OH group linked to the methylene (–CH_2_) group in KA was situated in proximity to the copper atoms, which is similar to the reported molecular docking simulation between KA and tyrosinase^53^^,^^55^.

Interestingly, the chlorine (–Cl) atom of compound **4** interacted with both CuA and CuB atoms, as well as catalytic histidine residues (H61, H85, H94, H259, and H296). In the case of KA, its –OH group bound to CuA and CuB and interacted with three histidine residues (H61, H259, and H296). These results suggested that the –Cl moiety of compound **4** plays a crucial role in stabilising its binding, potentially enhancing its affinity and inhibitory activity against tyrosinase.

The molecular docking results of 15 *bis*-thiourea compounds (Figures S18–S20) revealed interaction energies ranging from −5.6 to −7.4 kcal/mol. The 2D interaction profiles of these compounds indicated their ability to interact with the catalytic histidine residues, copper atoms, and amino acid residues located within the enzyme’s active site.

### 3.4. Structural stability

The stability of each simulated complex was assessed through the calculation of the RMSD for the ligand–tyrosinase complexes, along with the analysis of the Rg and #Contacts. Figure 5 illustrated that the RMSD values of both compound **4** and KA in complexes with tyrosinase reached equilibrium after 30 ns, maintaining the fluctuations of ∼2.50 Å until the conclusion of the simulations. It is noteworthy that compound **4** exhibited a lower RMSD value (2.56 ± 0.09 Å, calculated from the last 10 ns) compared to the KA system (2.69 ± 0.13 Å). Supportively, the Rg value of the compound **4**-tyrosinase complex (∼20.66 ± 0.05) was lower than that of the KA system (∼20.72 ± 0.06), suggesting a higher level of compactness in the complexation between compound **4** and tyrosinase. Furthermore, the #Contacts within 4 Å at the active site of tyrosinase for compound **4** (∼60.49 ± 10.08) was higher than that for the KA (∼44.61 ± 5.01), implying that compound **4** exhibited superior binding affinity to tyrosinase, as discussed later in the binding affinity analysis (Table 3).

### 3.5. Predicted binding affinity

The MM/PBSA-based Δ*G*_bind_ and its energy components were calculated using 100 snapshots extracted from the last 10 ns simulations. The obtained results are summarised in Table 3. The molecular mechanics (Δ*E*_MM_) energy in the gas phase revealed that the Δ*E*_vdW_ contribution was found to be favourable for compound **4** and KA in complexes with tyrosinase (–36.73 ± 0.44 and −16.47 ± 0.32 kcal/mol, respectively). Notably, the Δ*E*_MM_ value of compound **4**-tyrosinase was lower than that of the KA-tyrosinase system. The vdW-driven complexation between compound **4** and tyrosinase agrees well with the binding of bromophenols^56^, kuwanon G, mulberrofuran G, albanol B^54^, 2-phenyl-1,4-naphthoquinones^57^, and *bis*(4-hydroxybenzyl)sulfide^58^ to the tyrosinase enzyme.

The predicted Δ*G*_bind_ values of compound **4**-tyrosinase (–34.72 ± 0.45 kcal/mol) and KA-tyrosinase (–15.44 ± 0.38 kcal/mol) complexes were in good agreement with the experimental tyrosinase inhibitory activity results (Figure 3; Table 2), and supported by the results of structural stability, the compactness of the ligand–protein complex, and #Contacts (Figure 5) as mentioned above. These findings suggested that the binding affinity of compound **4**-tyrosinase complex was higher than that of KA-tyrosinase complex.

### 3.6. H-bond formations and key binding residues

**Figure 6.**
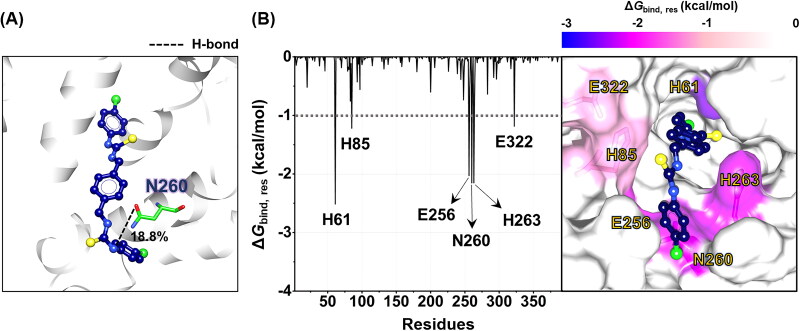
**(A)** The percentage of H-bond occupation of the amino acid residue within the active site of tyrosinase contributing to the binding of compound **4**. **(B)** (Left) Δ*G*_bind_, _res_ of compound **4** in complex with tyrosinase. The copper ions were not shown. (Right) Representative structures showing the orientation of compound **4** in the catalytic site drawn from the last MD snapshot. The residues involved in the ligand binding (energy stabilisation of ≤–1.0 kcal/mol) were coloured according to their Δ*G*_bind_, _res_ values, where the highest to lowest Δ*G*_bind_, _res_ values were shaded from white to blue, respectively.

H-bond represents one of the non-covalent interactions that impacts the strength of protein-ligand complex^59^. In this work, H-bond interactions were calculated using a distance of ≤3.5 Å between the hydrogen donor (HBD) and hydrogen acceptor (HBA), with an angle of ≥120° for HBD–H···HBA. The analysis of H-bond occupation was conducted during the final 10 ns of the MD simulation. Figure 6A illustrated that only the N260 residue of tyrosinase can form H-bond with the compound **4** (18.8%). This finding suggested that intermolecular H-bonding did not play a significant role in the complexation between compound **4** and tyrosinase, as supported by the Δ*E*_MM_ calculation (Table 3), and consistent with the binding patterns observed for Thiamidol^TM^^60^ and KA^53^. In contrast, the binding of compound **4** to the catalytic centre of tyrosinase was primarily driven by vdW interactions, as discussed above (Table 3).

We further explored the essential residues involved in the binding of compound **4** to tyrosinase using Δ*G*_bind, res_ calculations based on the MM/PBSA method. The energy contribution from each amino acid residue is shown in Figure 6B. Note that only residues displaying energy stabilisation of ≤ −1.0 kcal/mol were considered. Results highlighted six key residues, including H61, H85, E256, N260, H263, and E322, for the binding of compound **4**. It should be noted that three catalytic histidine residues (H61, H85, and H263) were involved in the complexation of compound **4**. The aforementioned residues were also found in the previously reported binding patterns of 2-phenyl-1,4-naphthoquinones^57^, epicatechin^61^, 5-O-β-d-glucopyranoside^53^, Thiamidol^TM^^60^, and benzimidazothiazolone derivatives^62^ against tyrosinase.

### 3.7. Copper-chelating activity

Copper plays a critical role in the activity of copper-dependent enzymes^44^, including tyrosinase, which is involved in the production of melanin. In this study, we evaluated the copper-chelating potential of compound **4**, comparing its activity to the known chelator, ethylenediaminetetraacetic acid (EDTA) and the known tyrosinase inhibitor, KA to assess its potential as a tyrosinase inhibitor.

**Figure 7.**
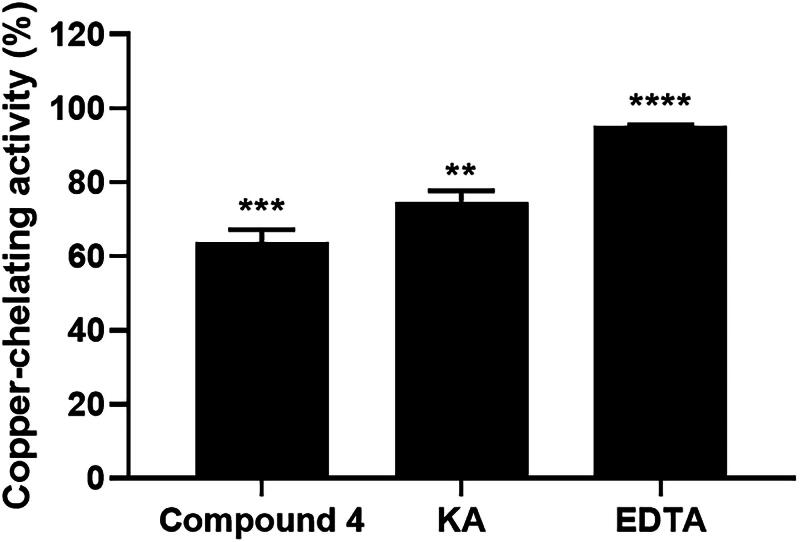
The copper-chelating activity of compound **4**, KA, and EDTA at 5 mM. Data are shown as the mean ± SEM (*n* = 3). **p < 0.01, ***p < 0.001, ****p < 0.0001 vs. control.

As shown in Figure 7, compound **4** at 5 mM demonstrated significant copper-chelating activity (64%), while that of KA and EDTA at 5 mM was 75% and 95%, respectively. This indicates that compound **4** is capable of interacting with copper ions, which are key cofactors of tyrosinase. This result is in good agreement with the molecular docking analysis (Figure 4) demonstrating that compound **4** can interact with copper atoms at the active site of tyrosinase, further supporting its potential as a tyrosinase inhibitor.

Previous studies have shown that various sulfur-containing compounds possess copper-binding capability. The 5,6,7,8-tetrahydro-4H-furo[3,2-c]azepine-4-thione (T4FAT) at 200 mM was capable of chelating more than 80% of copper ions^63^. Additionally, 2-thiobenzothiazole (2-TBT) showed copper-chelating activity ranging from approximately 48% to 69%^64^. These findings support our *bis*-thiourea **4** as a copper-chelating agent.

### 3.8. Cell viability

**Figure 8.**
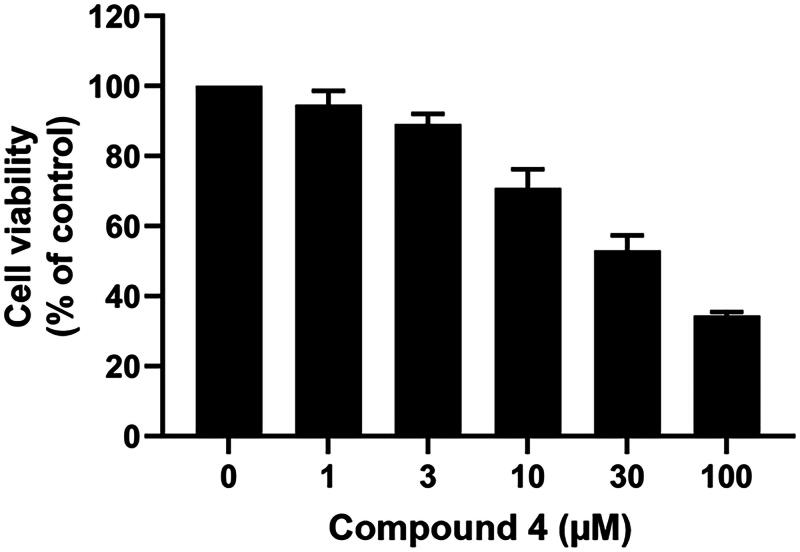
Cell viability of B16F10 cell treated with compound **4** at various concentrations for 48 h. Data are shown as the mean ± SEM (*n* = 3).

To evaluate the cytotoxic activity of compound **4**, B16F10 murine melanoma cells were treated with various concentrations of compound **4** for 48 h, followed by assessment of cell viability using the MTT assay. As illustrated in Figure 8, compound **4** exhibited dose-dependent reduction in B16F10 cell viability. Compound **4** caused strong cytotoxic effect at a concentration of 100 μM (cell viability of < 40%) on B16F10 cells. Therefore, the concentrations of compound **4** at 1 and 3 μM (cell viability of ∼90%), 10 μM (∼75%), and 30 μM (∼50%) were chosen for the further experiments.

### 3.9. Compound 4 inhibited melanin production in α-MSH-stimulated B16F10 murine melanoma cells

**Figure 9.**
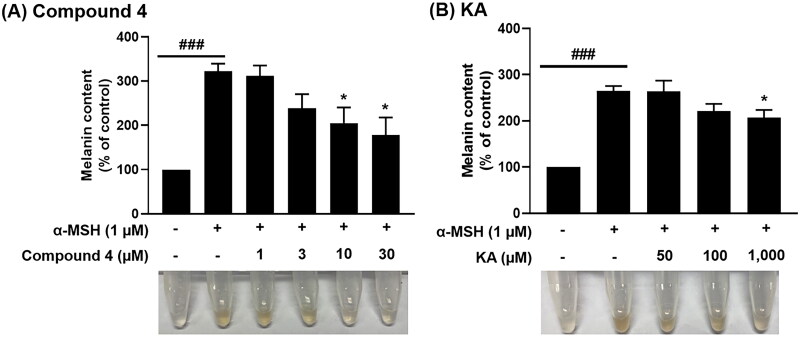
The anti-melanogenic effect of **(A)** compound **4** and **(B)** KA on B16F10 murine melanoma cells. Data are shown as the mean ± SEM (*n* = 3). ^###^*p* < 0.001 vs. control group. *p < 0.05 vs. α-MSH-treated group.

To assess the inhibitory effect of compound **4** on melanin synthesis, we measured melanin content in α-MSH-stimulated B16F10 murine melanoma cells treated with various concentrations of the test compounds for 48 h. As shown in Figure 9, α-MSH treatment significantly increased melanin production compared to the untreated control cells^65^. The KA, known tyrosinase inhibitor, reduced melanin content significantly only at a concentration of 1000 μM when compared to the α-MSH group. Notably, compound **4** significantly inhibited melanin production in α-MSH-treated B16F10 cells at concentrations of 10 and 30 μM. These findings indicated that compound **4** (Figure 9A) exhibits significantly stronger anti-melanogenic activity compared to KA (Figure 9B) by approximately 30–100 times.

This observation aligns well with the binding affinity results obtained from (i) molecular docking (Figure 4), (ii) Δ*G*_bind_ calculation based on MM/PBSA method (Table 3) and (iii) structural stability analysis (Figure 5). Collectively, these computational and experimental results affirmed that compound **4** could act as a potent tyrosinase inhibitor with notable anti-melanogenic activity.

### 3.10. Drug-likeness prediction

Prediction of drug-likeness is a crucial step in drug development^66^. In this study, we utilised the SwissADME web tool^43^, which calculates key drug-like properties, pharmacokinetics, physicochemical parameters, and related characteristics. Moreover, it provides fast predictive models demonstrating statistical significance, intuitive interpretation, and a straightforward conversion to molecular design^43^. To predict the drug-likeness of the potent *bis*-thiourea **4** in comparison with the KA, various parameters, including molecular weight (MW), the number of hydrogen bond donors and acceptors (HBD and HBA), the number of rotatable bonds (RB), topological polar surface area (TPSA), and lipophilicity (log *P*), were investigated. As shown in Table 4, the predicted values for compound **4** and KA adhered to Lipinski’s rule of five criteria^67^^,^^68^: (i) MW ≤ 500 Da, (ii) HBD ≤ 5 and HBA ≤ 10, (iii) RB ≤ 10, (iv) TPSA ≤ 140 Å^2^, and (v) log *P* ≤ 5. In addition, most of our *bis*-thiourea derivatives (Table S1) demonstrated drug-like properties, suggesting their potential for development as tyrosinase inhibitors.

**Table 4.** Predicted drug-likeness according to Lipinski’s rule of five criteria for compound **4** and KA.

Compound	Lipinski’s rule of five						
	MW (≤500 Da)	HBD (≤5)	HBA (≤10)	RB (≤10)	TPSA (≤140 Å²)	Log *P* (≤5)	Drug-likeness
**4**	475.46	4	0	10	112.30	4.50	Yes
KA	142.11	2	4	1	70.67	–1.69	Yes

## 4. Conclusion

This study employed *in vitro* and *in silico* approaches to evaluate the inhibitory activity of *bis*-thioureas against mushroom tyrosinase. Among the 16 synthesised derivatives, compound **4** showed the highest anti-tyrosinase activity. Furthermore, compound **4** in complex with tyrosinase demonstrated higher structural stability than KA-tyrosinase complex. The MM/PBSA-based Δ*G*_bind_ calculation indicated that compound **4-**tyrosinase complex exhibited the higher binding affinity compared to KA-tyrosinase system, and vdW forces were identified as the main interaction for complexation. The N260 residue of tyrosinase could form H-bond with compound **4**. H61, H85, E256, N260, H263, and E322 residues were suggested as hotspot residues for the binding of compound **4** to tyrosinase. Copper-chelating activity assay confirmed that compound **4** could chelate the copper ions. Moreover, melanin assay demonstrated that compound **4** markedly reduced melanin production in B16F10 murine melanoma cells, displaying significantly stronger anti-melanogenic activity compared to KA. The drug-likeness prediction supports the potential of compound **4** as a promising lead for the development of novel tyrosinase inhibitors based on *bis*-thiourea scaffolds.

## Supplementary Material

Supplementary data anonymous.docx

## Data Availability

The authors confirm that the data supporting the findings of this study are available within the article and/or its supplementary materials.
