# Supplementary material for: Discovery of bis-thiourea derivatives as potent tyrosinase inhibitors: combined experimental and computational study
Source: J Enzyme Inhib Med Chem. 2025 Jun 24;40(1):2518195. doi: 10.1080/14756366.2025.2518195 (PMC12897533; doi:10.1080/14756366.2025.2518195)
Supplement: Supplementary data anonymous.docx [file IENZ_A_2518195_SM8736.docx]

**Supplementary information**

**Discovery of *Bis*-thiourea Derivatives as Potent Tyrosinase Inhibitors: Combined experimental and computational study**

**Figure S1**. HPLC chromatogram of compound **1**.

**Figure S2**. HPLC chromatogram of compound **2**.

**Figure S3**. HPLC chromatogram of compound **3**.

**Figure S4**. HPLC chromatogram of compound **4**.

**Figure S5**. HPLC chromatogram of compound **5**.

**Figure S6**. HPLC chromatogram of compound **6**.

**Figure S7**. HPLC chromatogram of compound **7**.

**Figure S8**. HPLC chromatogram of compound **8**.

**Figure S9**. HPLC chromatogram of compound **9**.

**Figure S10**. HPLC chromatogram of compound **10**.

**Figure S11**. HPLC chromatogram of compound **11**.

**Figure S12**. HPLC chromatogram of compound **12**.

**Figure S13**. HPLC chromatogram of compound **13**.

**Figure S14**. HPLC chromatogram of compound **14**.

**Figure S15**. HPLC chromatogram of compound **15**.

**Figure S16**. HPLC chromatogram of compound **16**.

**
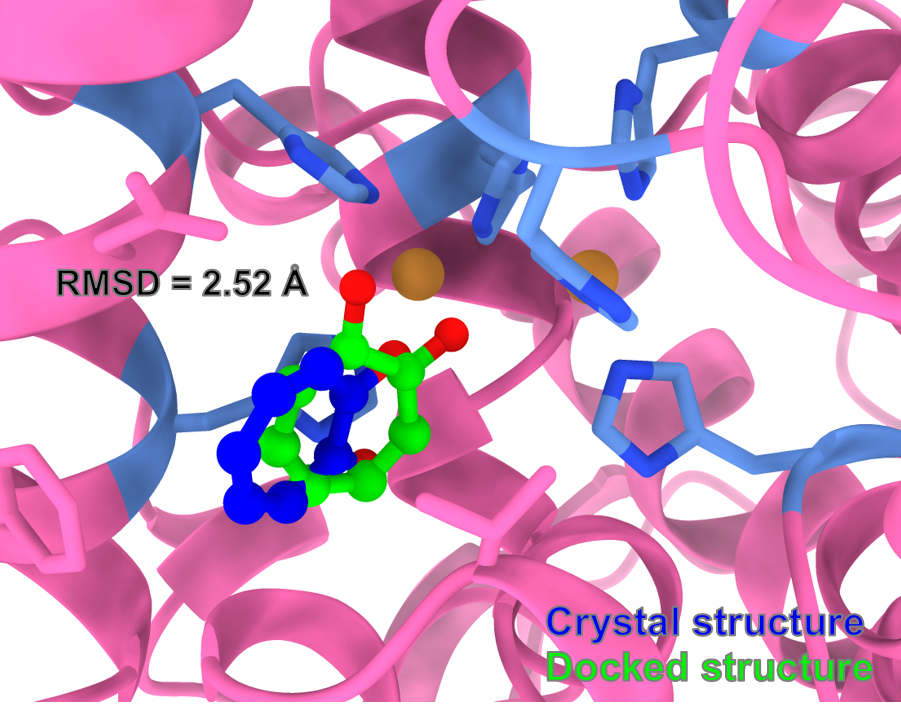
**

**Figure S17.** Superimposition between the crystalized tropolone (blue) (PDB ID: 2Y9X^15^) and the docked tropolone (green) obtained from the CB-DOCK2 web server.


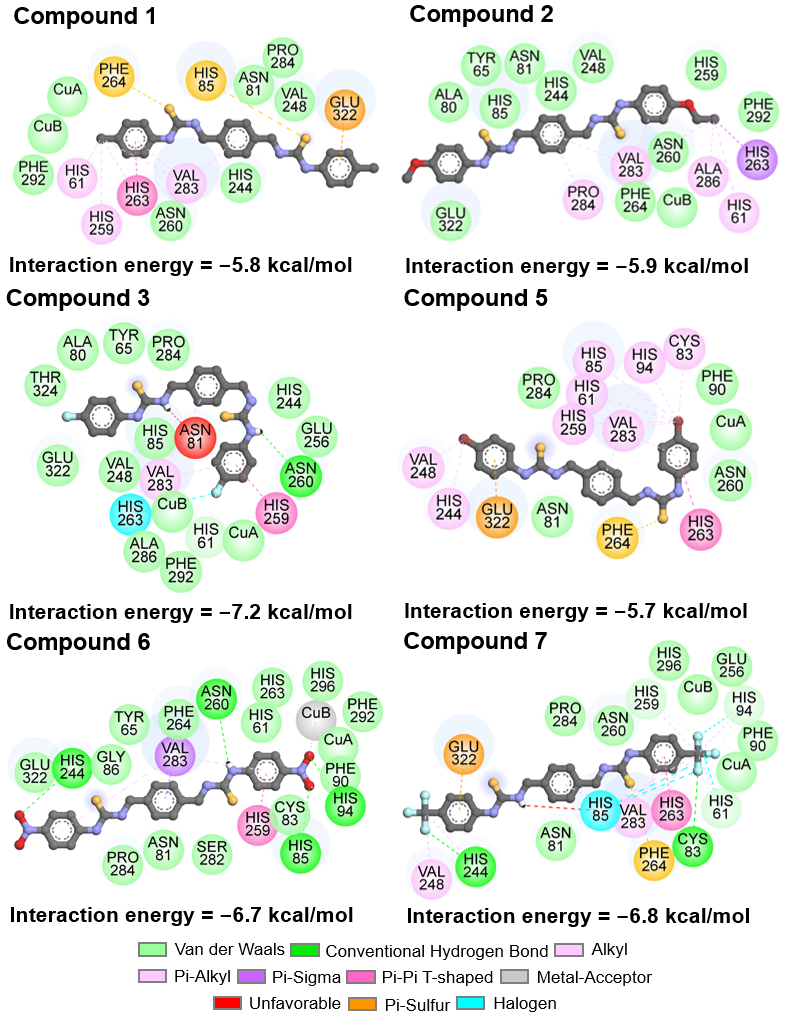


**Figure S18**. 2D interaction profile of compounds **1, 2, 3, 5, 6** and **7** in complexes with tyrosinase obtained from molecular docking.


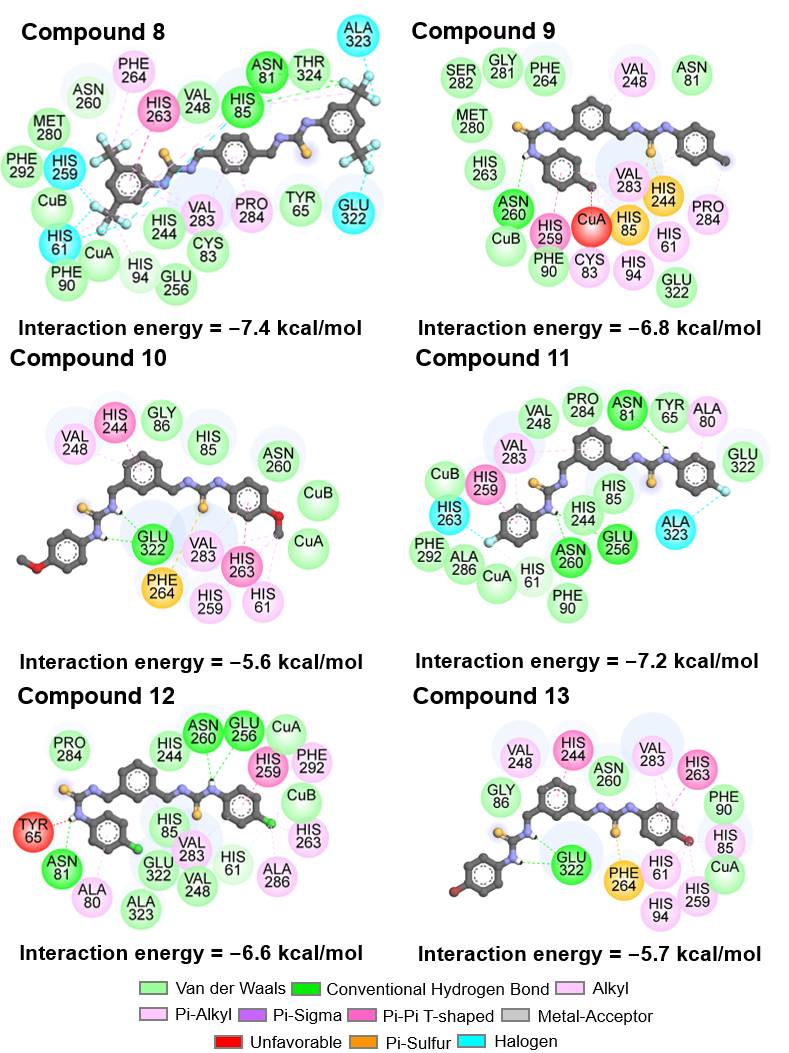


**Figure S19**. 2D interaction profile of compounds **8**-**13** in complexes with tyrosinase obtained from molecular docking.


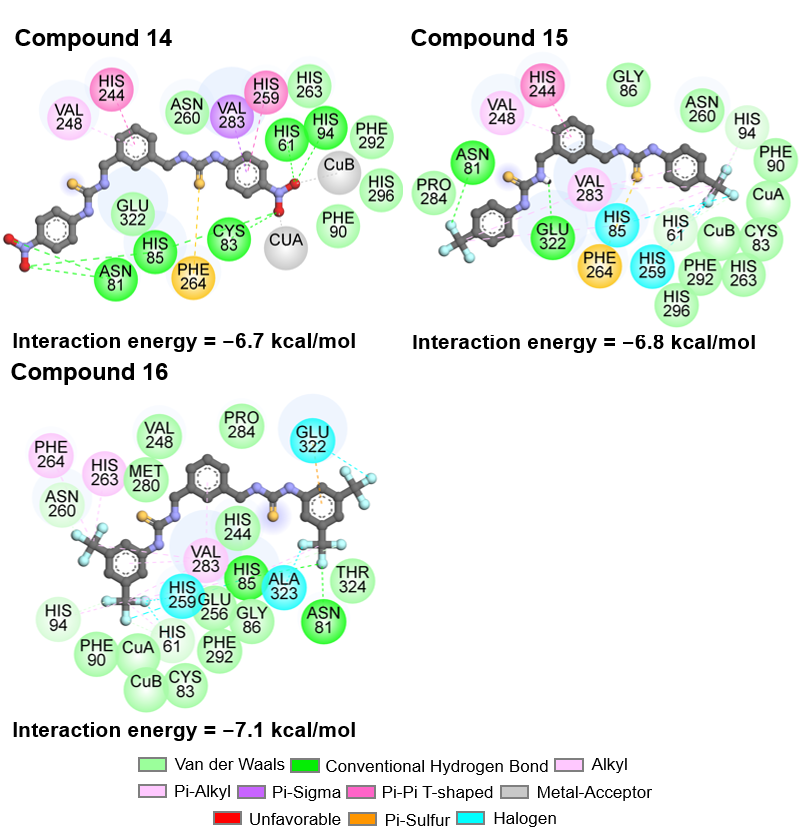


**Figure S20**. 2D interaction profile of compounds **14**-**16** in complexes with tyrosinase obtained from molecular docking.

**Table S1.** Predicted drug-likeness according to Lipinski's Rule of Five criteria for 15 *bis*-thiourea derivatives.

| **Compound** | **Lipinski’s rule of five** | | | | | | |
| --- | --- | --- | --- | --- | --- | --- | --- |
|  | **MW (≤500 Da)** | **HBD (≤5)** | **HBA (≤10)** | **RB**  **(≤10)** | **TPSA**  **(≤140 Å²)** | **Log *P***  **(≤5)** | **Drug-likeness** |
| **1** | 434.62 | 4 | 0 | 10 | 112.30 | 3.96 | Yes |
| **2** | 466.62 | 4 | 2 | 12 | 130.76 | 2.86 | Yes |
| **3** | 442.55 | 4 | 2 | 10 | 112.30 | 4.29 | Yes |
| **5** | 564.36 | 4 | 0 | 10 | 112.30 | 4.71 | No |
| **6** | 496.56 | 4 | 4 | 12 | 203.94 | 2.54 | Yes |
| **7** | 542.56 | 4 | 6 | 12 | 112.30 | 5.11 | No |
| **8** | 678.56 | 4 | 12 | 14 | 112.30 | 6.08 | No |
| **9** | 434.62 | 4 | 0 | 10 | 112.30 | 3.96 | Yes |
| **10** | 466.62 | 4 | 2 | 12 | 130.76 | 2.86 | Yes |
| **11** | 442.55 | 4 | 2 | 10 | 112.30 | 4.29 | Yes |
| **12** | 475.46 | 4 | 0 | 10 | 112.30 | 4.50 | Yes |
| **13** | 564.36 | 4 | 0 | 10 | 112.30 | 4.71 | No |
| **14** | 496.56 | 4 | 4 | 12 | 203.94 | 2.54 | Yes |
| **15** | 542.56 | 4 | 6 | 12 | 112.30 | 5.11 | No |
| **16** | 678.56 | 4 | 12 | 14 | 112.30 | 6.08 | No |
